# The disease burden of congenital toxoplasmosis in Denmark, 2014

**DOI:** 10.1371/journal.pone.0178282

**Published:** 2017-05-30

**Authors:** Janna Nissen, Pikka Jokelainen, Christen Rune Stensvold, Chiara Trevisan, Josefine Fuchs, Kristoffer Sølvsten Burgdorf, Henrik Vedel Nielsen, Sara M. Pires

**Affiliations:** 1 Division of Diet, Disease Prevention and Toxicology, National Food Institute, Technical University of Denmark, Lyngby, Denmark; 2 Laboratory for Parasitology, Statens Serum Institut, Copenhagen, Denmark; 3 Faculty of Veterinary Medicine, University of Helsinki, Helsinki, Finland; 4 Department of Basic Veterinary Sciences and Population Medicine, Institute of Veterinary Medicine and Animal Science, Estonian University of Life Sciences, Tartu, Estonia; 5 Department of Veterinary Disease Biology, University of Copenhagen, Frederiksberg, Denmark; 6 Department of Ophthalmology, Copenhagen University Hospital, Glostrup, Denmark; 7 Department of Clinical Immunology, Copenhagen University Hospital, Copenhagen, Denmark; Université Catholique de Louvain, BELGIUM

## Abstract

**Background:**

Congenital toxoplasmosis (CT) causes a substantial disease burden worldwide. The aim of this study was to estimate the disease burden of CT in Denmark, a developed country with free public healthcare and nationwide data available.

**Methods:**

Using data primarily from two public health surveillance programmes conducted between 1992 and 2007, we estimated the incidence, occurrence of sequelae, mortality and the burden of disease in terms of disability-adjusted life years (DALYs) of CT in Denmark in 2014.

**Findings:**

We estimated that 14 children were born with CT in 2014, of which six will have developed sequelae by the age of 12. CT resulted in a total disease burden of 123 DALYs (95% uncertainty interval [UI], 100–148), of which 78 (95% UI, 64–94) were due to foetal loss and 2 (95% UI, 1–3) were due to neonatal death; the remaining burden was due to moderate to severe life-long sequelae. A comparison of the estimated incidence of CT with the number of reported CT cases in 2008–2014 indicated that for each reported CT case, at least five other CT cases could be expected to have occurred and gone unreported.

**Interpretation:**

Early onset, severity, and life-long duration of sequelae have a major effect on the disease burden of CT. Our data suggest that CT is under-diagnosed or under-reported in Denmark. The estimated disease burden and public health impact in Denmark is lower than in other European countries, highlighting the need for country-specific studies.

## Introduction

Toxoplasmosis is a zoonotic disease. Humans can acquire *Toxoplasma gondii* infection through foodborne or environmental exposure. Recent estimates suggest that more than one million cases of foodborne toxoplasmosis occur in Europe annually [1]. Infection of pregnant women by *T*. *gondii* is particularly problematic, as the parasite may cross the placenta and infect the unborn child, resulting in congenital toxoplasmosis (CT) [2]. CT may lead to miscarriage, stillbirth or sequelae in the child; however, the majority of congenitally infected children appear asymptomatic at birth. The maternal-foetal transmission rate upon primary infection increases with gestational age, ranging from 6% (95% confidence interval [CI], 3–9) at 13 weeks of gestation to 72% (95% CI, 60–81) at 36 weeks of gestation [2]. The earlier the foetus is infected, the higher the risk of developing severe sequelae [2]. The typical clinical signs of CT include chorioretinitis, intracranial calcification, hydrocephalus and CNS abnormalities leading to neurological deficiencies (psychomotor or other neurological deficiencies, convulsions, and mental retardation). Furthermore, CT may cause foetal loss and neonatal death [3]. In contrast, acquired *T*. *gondii* infection will in most cases run a subclinical course or cause minor symptoms (fever, fatigue, lymphadenopathy); nevertheless, some individuals may develop ocular or generalized toxoplasmosis [4].

Burden of disease studies have been recognized as a powerful tool to enable policy makers and other stakeholders to set appropriate, evidence-based priorities for food safety interventions [5]. A recent World Health Organization (WHO) initiative to estimate the regional and global burden of foodborne diseases (Foodborne Disease Burden Epidemiology Reference Group, FERG) calculated combined disease burden estimates for congenital and acquired toxoplasmosis in Europe, ranking *T*. *gondii* the third most important cause of foodborne disease [1]. WHO—FERG highlighted the need for national studies to achieve more accurate and country-specific estimates. However, only a few countries have so far been able to accomplish this task. In The Netherlands, the disease burden caused by toxoplasmosis ranked highest among 14 foodborne diseases and fifth among 32 infectious diseases [6,7], largely explained by the burden of CT. In Greece, toxoplasmosis ranked fifth among 19 foodborne diseases [8]. In the United States, toxoplasmosis ranked second among seven leading foodborne pathogens mainly due to the burden of acquired toxoplasmosis [9]. The burden of CT has also been estimated in low-income countries such as Nepal [10] and Kyrgyzstan [11]. All these studies identified substantial data gaps due to the lack of effective surveillance of the disease at the national level.

In this study, we estimated the disease burden of CT in 2014 in Denmark—a developed country with a free public healthcare system, a history of active CT-screening of newborns, and nationwide data available. In Denmark, one regional study and one national study on CT have been conducted [12,13]. We estimated the national incidence, occurrence of sequelae, mortality, and the burden of CT in terms of disability-adjusted life years (DALYs), a metric that enables an objective and complete comparison between diseases and risk factors [14,15]. Furthermore, we compared the estimated incidence of CT in Denmark with the number of CT cases captured by surveillance during a period with no active screening to assess the current level of under-reporting of the disease.

## Materials and methods

DALYs reflect the sum of the number of years lived with disability (YLDs) and the number of years of life lost (YLLs) due to premature mortality. YLDs are estimated by multiplying the number of incident cases with the duration and the disability weight (DW) of a health state, while YLLs are estimated by multiplying the number of deaths and life expectancy at the age of death [16]. DWs express the proportional reduction of quality of life due to sequelae on a scale from 0 to 1, where 0 means perfect health and 1 equals death [17], and were obtained from the Global Burden of Disease 2013 study [15] and Havelaar et al (2007) [18]. We calculated DALYs for 2014, and used the Danish life expectancy table [19] to estimate YLDs and the frontier national life expectancy projected for the year 2050 by the World Population Prospects 2012 (UN Population Division, 2013) [20] to estimate YLLs.

### Health outcomes

**Fig 1:**
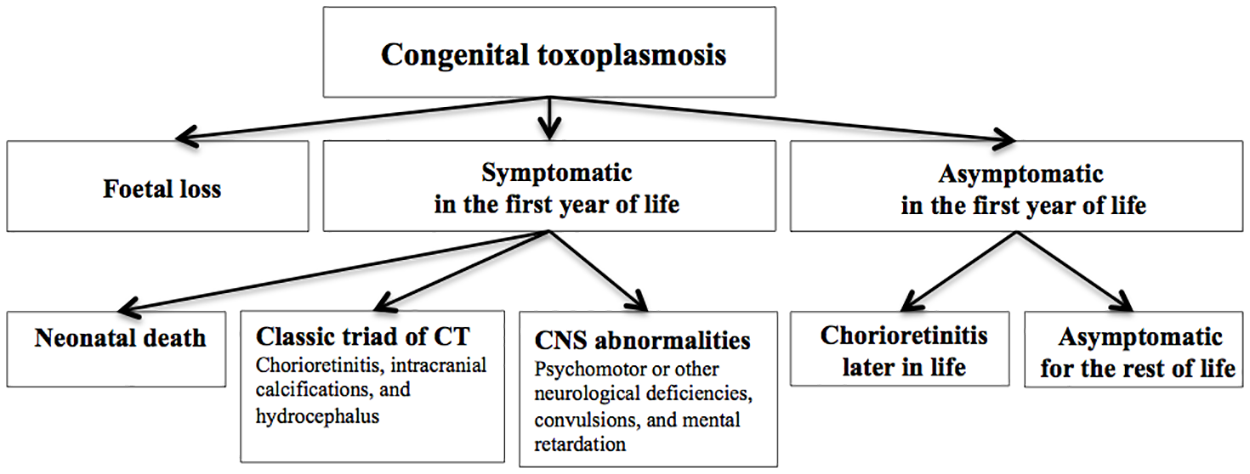
Health outcome tree for congenital toxoplasmosis (CT), adapted from Havelaar et al. (2007) [18].

CT comprises a variety of health outcomes that occur after congenital infection with *T*. *gondii* (Fig 1). In this paper, we use the expression CT also for children with no apparent symptoms or signs at birth due to the congenital *T*. *gondii* infection so as to include all possible outcomes in the disease burden estimate. Health outcomes of CT were defined according to Havelaar et al. (2007) [18]: including foetal loss, sequelae manifesting in the first year of life (chorioretinitis, intracranial calcification, hydrocephalus, CNS abnormalities, and neonatal death), and sequelae appearing after the first year of life (chorioretinitis) (Fig 1). Non-specific symptoms following CT (e.g., anaemia, jaundice, pneumonitis, and diarrhoea) [21] were not considered. CT was considered possible for infants of previously seronegative mothers who became infected during pregnancy; the possibility of periconceptional transmission or vertical transmission in mothers deemed seropositive prior to conception was not considered.

### Data sources

During the Danish Neonatal Feasibility Study (DNFS), running from June 1992 to August 1996 [12], pregnant women from five counties in Denmark who gave birth to live infants, representing one third of all deliveries in the country, were offered screening at delivery for primary *T*. *gondii* infection acquired during pregnancy and for CT. Guthrie cards of newborns were analysed for *T*. *gondii*-specific IgG antibodies (DAKO, Glostrup, Denmark) and during the last 12 months of the study also for *T*. *gondii*-specific IgM antibodies by enzyme immunosorbent assay (EIA). Furthermore, Guthrie cards from *T*. *gondii*-infected newborns identified before September 1, 1995, were also analysed for *T*. *gondii*-specific IgM antibodies after being stored for up to three years. When *T*. *gondii*-specific IgG or IgM antibodies were detected on a Guthrie card, the mother’s first-trimester serum sample (routinely taken for syphilis testing at weeks 8 to 12 of gestation) was thawed and analysed for *T*. *gondii*-specific IgG antibodies. It was considered evidence of maternal seroconversion if *T*. *gondii*-specific IgG or IgM antibodies were detected on the newborns Guthrie card and the mother’s first trimester sample was negative. First-trimester samples that were negative for IgG by EIA were subsequently analysed by the Sabin—Feldman dye test. When CT was suspected, confirmatory diagnostic serology was performed on blood samples from mother and child within 6 weeks after birth [12].

Of 89,873 children sampled during the DNFS, 27 children were diagnosed with CT, corresponding to an apparent incidence of 3.0 per 10,000 live-born children. Follow-up was performed for 26 of the 27 children for late development of chorioretinitis with a maximum follow-up time of 12 years (4–12 years; median age, 10 years) (https://figshare.com/s/ca8e1a41aeac216204f1).

The Danish National Neonatal Screening Programme for Congenital Toxoplasmosis (DNNSP), running from January 1999 to July 2007 [22,23], included > 98% of all newborns in Denmark. Pregnant women were not screened during the program. Guthrie cards of newborns were analysed for *T*. *gondii*-specific IgM and IgA antibodies by fluorescence enzyme immunoassay (FEIA) or time-resolved immunofluorometric assay (TRIFMA). If positive, Guthrie cards were further analysed by a modified immunosorbent agglutination assay (ISAGA; bioMérieux, France) for *T*. *gondii*-specific IgM antibodies. When CT was suspected (Guthrie card ISAGA IgM-positive), serum samples from both mother and child were analysed for *T*. *gondii*-specific IgG and IgM antibodies by the Vidas system (bioMérieux, France), for IgA antibodies by the Platelia system (PasteurDiagnostics, Paris), for *Toxoplasma*-specific antibodies by the Sabin—Feldman’s dye test (in-house, Statens Serum Institut, Copenhagen, Denmark), and for *Toxoplasma*-specific IgM by ISAGA (bioMérieux, France) [23]. At one year of age, children diagnosed with CT at birth had a new serum sample analysed for *T*. *gondii*-specific IgG antibodies for serological confirmation. Active surveillance of CT in Denmark was terminated in 2007 [13].

Of the approximately 560,000 children born during the DNNSP, 105 children were diagnosed with CT, corresponding to an apparent incidence of 1.9 per 10,000 live-born children. The 55 children born with CT in the first four years of the DNNSP were followed for three years for developmental and clinical outcomes [23]; these data were used to estimate sequelae in the first year of life.

### Incidence of CT and related health outcomes

The incidence of CT in newborns appeared stable during the DNNSP, and hence we assumed it was applicable to the reference year 2014.

To estimate the incidence of CT and the selected associated health outcomes of CT for 2014, we defined a set of parameters (Table 1). We used the number of diagnosed CT cases by DNNSP and accounted for the sensitivity of the diagnostic approach (largely affected by detection of *T*. *gondii*-specific IgM antibodies on Guthrie card; 70%-80% [12]) to estimate the number of children born with CT. The proportion of children diagnosed with chorioretinitis, intracranial calcification, hydrocephalus, and CNS abnormalities were derived from the first four years of the DNNSP [22,23], and the proportion of children who were asymptomatic in the first year of life and data on the occurrence of chorioretinitis later in childhood were derived from a follow-up study of the DNFS.

**Table 1:** Parameters used to estimate the incidence of congenital toxoplasmosis (CT) and selected associated health outcomes.

Notation	Description	Value/Distribution/Estimation	Reference
Lb	Number of live births in 2014	56,870	Statistics Denmark, accessed on 18 April, 2016
Sb	Stillbirths after gestational week 22 in 2014	234	Statistics Denmark, accessed on 18 April, 2016
Sa	Number of reported spontaneous abortions occurring up to 22 weeks of gestation	11,928	[24]
Tp	Total number of pregnancies	Lb + Sb + Sa = 69,104	-
PSeroNeg	Proportion of seronegative pregnant women	RiskBeta (3925;1479)	[25]
PSeroConv	Probability of seroconversion during pregnancy	RiskBeta (140;64,746)	DNFS
Seroconv	Number of seroconverting pregnant women	Tp* PSeroNeg* PSeroConv	-
PToxoAb	Probability of fetal loss in seroconverting mothers	RiskBeta (48;2,630)	[26]
FoetalLoss	Number of foetal losses due to CT >22 weeks of gestation	Seroconv* PToxoAb*((40–22)/40)	-
IncCT	Annual incidence of CT diagnosed in the first year of life (cases per 10,000)	1.9	DNNSP
Sens	Sensitivity of the diagnostic approach	1/RiskUniform (0.7–0.8)	DNFS
CasesCT	Total cases of postnatal CT	(Lb x Inc CT)/Sens	-
ProbCS1	Probability of developing sequelae in the first year of life	RiskBeta (13;44)	DNNSP
ProbCh1	Probability of developing chorioretinitis	RiskBeta (8;6)	DNNSP
ProbIC	Probability of developing intracranial calcifications	RiskBeta (11;3)	DNNSP
ProbHC	Probability of developing hydrocephalus	RiskBeta (2;12)	DNNSP
ProbCNS	Probability of developing CNS abnormalities	RiskBeta (2;12)	DNNSP
ProbND	Probability of neonatal death	RiskPert (0;0.007;0.012)*	DNFS, DNNSP and [18]
ProbCS2	Probability of being asymptomatic in the first year of life	1-ProbCS1	DNFS
ProbCh2	Probability of developing chorioretinitis later in life (1 to 12 years of age)	RiskBeta (7;18)	DNFS

DNFS: The Danish Neonatal Feasibility Study [12]; DNNSP: The Danish National Neonatal Screening Programme for Congenital Toxoplasmosis [13,22,23]

* Minimum value informed by Danish data (zero neonatal deaths reported in DNFS and DNNSP); most likely and maximum value adapted from Havelaar et al. (2007) [18]

Initially, we calculated the total number of pregnancies in Denmark in 2014 by summing the number of live-born children, the number of stillbirths after 22 weeks of gestation, and the number of registered spontaneous abortions occurring up to 22 weeks of gestation [24]. Assuming that the proportion of women who were seronegative at the beginning of pregnancy (mean 72.63% ± 0.61 standard deviation [SD]), [25] and the rate of seroconversion of seronegative pregnant women (0.21%, 95% CI, 0.18–0.25) observed in the DNFS study [12] was applicable to 2014, we estimated the total number of pregnant women who seroconverted during pregnancy. Next, we estimated the number of pregnancies resulting in foetal loss >22 weeks of gestation in seroconverting women. Based on the methodology described by Havelaar et al. (2007) [18] we assumed that the probability of foetal loss in seroconverting mothers is 1.7% (mean 1.79% ± 0.26 SD) [26], and that foetal losses are generally equally distributed over the 40 weeks of gestation, giving an inclusion of 18 (i.e., 40–22 weeks)/40 of the estimated foetal losses due to CT in 2014.

To estimate the number of neonatal deaths, and in the absence of reports in Denmark, we adopted the most likely and maximum values of the probability of CT related neonatal death from Havelaar et al. (2007) [18] and defined zero as a minimum value for the probability as observed in DNFS and DNNSP.

All calculations of incidence of CT and of the development of health outcomes were performed using a stochastic model implemented in @risk 6.0 (Palisade Corporation, 2014).

### DALYs

To estimate DALYs, we combined incidence of morbidity and mortality estimates with data on the duration and DWs of the health outcomes, and life expectancy in the population. We assumed that children with chorioretinitis had, on average, moderately impaired distance vision [27] and used the updated DW of 0.031 for “moderate vision impairment” reported in the Global Burden of Disease study (GBD-2013) [15]. The DWs used for the neurological disorders were adopted from Havelaar et al. (2007) [18]. Only foetal loss >22 weeks of gestation was considered as a death, which consequently resulted in the loss of the full life expectancy (i.e., 92 years).

To estimate the associated uncertainty, we applied a stochastic model using the DALY Calculator interface developed in R (http://daly.cbra.be/).

### Scenario analysis

To estimate the impact of different model specifications and assumptions, we performed scenario analysis (SA) and evaluated the respective changes in the estimate of the disease burden of CT.

In SA1, the impact of ‘foetal loss’ was excluded from the baseline model, because losing a foetus may not lead to sequelae in the mother, who might soon become pregnant again. In SA2, we used different DWs for chorioretinitis, ranging from 0 to 0.2, to assess the impact of different degrees of visual impairment. The GBD-2013 study defined DWs for distance vision impairment ranging from 0.003 for mild impairment to 0.187 for severe impairment and blindness [15]. In SA3, we applied different combinations of non-uniform age weighting and 3% time-discount rate. Application of age weighting relies on the assumption that the life at different ages is unequally valued; e.g. youngest and oldest age is given less weight. By applying time discounting, future life-years are assigned less value than those lived today.

## Results

### Incidence of CT

The annual incidence rates during the DNNSP ranged between 1.2 and 2.9 cases of CT per 10,000 live-born children, with an overall diagnosed incidence rate of 1.9 cases per 10,000 live-born children for the period 1999–2007 [22,28] (Table 2). We estimated that 14 children (95% UI, 14–15) were born with CT in 2014. Of these, three children (95% UI, 2–5) were estimated to be born with CT related sequelae, three children (95% UI, 1–5) were estimated to be asymptomatic in the first year of life but to develop chorioretinitis by the age of 12, and eight children (95% UI, 6–10) were estimated to be asymptomatic by the age of 12.

**Table 2:** Incidence of congenital toxoplasmosis (CT) in Denmark from January 1, 1999, to July 31, 2007; cases per 10,000 live-born children.

Year	1999	2000	2001	2002	2003	2004	2005	2006	2007***	1999–2007***
Live-births in Denmark* (n)	66,220	67,084	65,458	64,075	64,599	64,609	64,282	67,131	36,689	560.147
Diagnosed with CT** (n)	11	13	19	12	13	8	9	14	6	105
**Incidence per 10,000**	1.7	1.9	2.9	1.9	2.0	1.2	1.4	2.1	1.6	1.9

* Statistics Denmark, accessed on 18 April, 2016 (http://www.danmarksstatistik.dk/en)

** The Danish National Neonatal Screening Programme for Congenital Toxoplasmosis (DNNSP).

*** DNNSP was terminated on July 31, 2007.

### Foetal loss

We estimated that 105 women (95% UI, 94–118) acquired a primary *T*. *gondii* infection during pregnancy in 2014, which resulted in an estimate of 0.84 (95% UI 0.6–1.0) CT-related foetal losses.

### Incidence of clinical health outcomes of CT

The estimated incidence of each health outcome of CT in 2014 is presented in Table 3. Among those with sequelae of CT in the first year of life, the most frequent outcomes were estimated to be intracranial calcification (3 cases; 95% UI, 1–4), and chorioretinitis (2 cases; 95% UI, 1–3). Hydrocephalus and CNS abnormalities were estimated to be less frequent (0.4 cases; 95% UI, 0–1). We estimated 0.02 neonatal deaths (95% UI, 0–0.03), and that three cases (95% UI, 1–5) of chorioretinitis would have occurred among the children born with asymptomatic CT in 2014 at some point between 1 and 12 years of age.

**Table 3:** Estimated incidence and disease burden of congenital toxoplasmosis (CT) in Denmark, 2014.

	Reported cases^1^ 1999–2002	Estimated cases		Duration years^2^	Disability weight (Mean and 95% UI)	Total DALYs^3^ (Median and 95% UI)
		Cases per 1,000 live births in 2014 (Median and 95% UI)	Total cases in 2014 (Median and 95% UI)			
Foetal loss ≥ 22 weeks of gestation	-	-	0.84 (0.6–1)	92	1	78 (64–94)
**Symptomatic *in the first year of life***						
Chorioretinitis	7	0.03 (0.01–0.06)	2 (1–3)	81	0.031 (0.019–0.049)	5 (3–8)
Intracranial calcification	10	0.04 0.02–0.07)	3 (1–4)	81	0.01*	2 (1–3)
Hydrocephalus	1	0.01 (0.001–0.02)	0.40 (0–1)	81	0.36 (0.16–0.56)	13 (4–29)
CNS abnormalities	1	0.01 (0.001–0.02)	0.40 (0–1)	81	0.36*	14 (5–27)
Neonatal death^4^	0	0.0004 (0.0001–0.001)	0.02 (0–0.03)	92	1	2 (1–3)
***Asymptomatic in the first year of life***						
Chorioretinitis later in life (Follow up to 12 years)^5^	6	0.05 (0.02–0.1)	3 (1–5)	69	0.031 (0.019–0.049)	7 (4–11)
Total	-	-	-	-	-	123 (100–148)

DALYs, disability-adjusted life years; UI, uncertainty interval.

^1^Number of cases reported from the initial four years (1999–2002) of the Danish National Neonatal Screening Programme for CT (DNNSP[22,23]), which included >98% of newborns

^2^Duration of all health outcomes is life-long. We used the Danish life expectancy table (www.danmarksstatistik.dk/en) to estimate YLDs and the frontier national life expectancy projected for the year 2050 by the World Population Prospects 2012 (UN Population Division, 2013) [20] to estimate YLLs.

^3^Calculated as median incidence x duration x disability weight

^4^Minimum data from Denmark (zero neonatal deaths reported in initial four years [1999–2002] of DNNSP [23]); most likely and high value adapted from Havelaar et al. (2007) [18], giving an interval of 0.7% (0–1.2)

^5^Chorioretinitis later in life (at the age of 4–12 years) based on follow-up observations of The Danish Neonatal Feasibility Study (DNFS).

* No uncertainty interval available.

### DALYs

The estimated disease burden of CT in Denmark in 2014 was 123 DALYs (95% UI, 100–148) (Table 3), with a total of 80 YLLs (95% UI, 66–96) and 42 YLDs (95% UI, 27–62). Foetal loss resulted in 78 DALYs (95% UI, 64–94), representing more than 50% of the total disease burden, and neonatal death resulted in 2 DALYs (95% UI, 1–3). Despite the low incidence, hydrocephalus and CNS abnormalities were estimated to cause 13 DALYs (95% UI, 4–29) and 14 DALYs (95% UI, 5–27), respectively; the remaining health outcomes caused a lower burden.

### Scenario analyses

Excluding the impact of foetal loss (SA1) reduced the overall burden of disease to 44 DALYs (95% UI, 28–64) or 4.89 DALYs per case (S1 Table). Assuming that all cases of chorioretinitis would be mild (i.e., DW = 0; SA2), the estimated disease burden per case was 1.1 times lower than the estimate of the baseline model (S1 Table). On the contrary, assuming that all cases of chorioretinitis would be severe (i.e., DW = 0.2; SA2), the estimated disease burden per case was 1.5 times higher than the estimate of the baseline model (S1 Table).

The analyses with different combinations of age weighting and time discounting (SA3) showed that if life is valued unequally at different stages of life (applying age weighting), the estimate of disease burden would be highest (14 DALYs per case) (S1 Table). Conversely, not including age weighting but assuming that future life years are assigned less value than those lived today (applying time discounting) yielded a lower estimate of disease burden (five DALYs per case) (S1 Table).

### Under-ascertainment of CT

Since the DNNSP was terminated in July 2007, between zero and five infants were registered with CT in Denmark annually (2008–2014) (Table 4). Comparing our estimated incidence with the number of registered CT cases suggests that for each reported CT case, at least five other CT cases could be expected to have occurred (95% UI, 2–7). Furthermore, our estimates show that even symptomatic CT cases could have been missed.

**Table 4:** Number of estimated (1) and registered (2) cases of congenital toxoplasmosis (CT) in Denmark in the period of 2008–2014.

Year	2008	2009	2010	2011	2012	2013	2014
Live-births in DK*	65,038	62,818	63,411	58,998	57,916	55,873	56,870
**1. Total estimated no. of CT cases**	16	16	16	15	15	14	14
No. of cases with sequelae in the first year of life	4	4	4	3	3	3	3
No. of cases with no sequelae in the first year of life but who will have developed chorioretinitis by the age of 12	3	3	3	3	3	3	3
No. of cases who will not have developed sequelae by the age of 12	9	9	9	8	8	8	8
2. No. of registered cases of CT**	5***	1	3	0	1	1	0

* Statistics Denmark, accessed on 1 June 2016 (http://www.danmarksstatistik.dk/en/)

** The Danish National Registry of Patients, accessed on 23 June 2016.

*** One of these cases was registered only in 2009.

## Discussion

We estimated that 14 children were born with CT in 2014, which corresponded to a total disease burden of 123 DALYs. Our results are in agreement but slightly higher than the findings by Torgerson and Mastroiacovo (2013) who estimated 13 CT (95% CI, 10–16) cases per year causing 82 DALYs (95% CI, 44–137) for Denmark [27]. In our model, most of the years of life lost were due to foetal losses and to the occurrence of relatively severe and life-long sequelae in children since birth. The clinical manifestations resulting in the highest number of healthy life years lost were estimated to be hydrocephalus and CNS abnormalities.

The main strength of this study was that regional and nationwide data were available for estimating the disease burden of CT at the national level. Because the DNNSP was a national surveillance programme including more than 98% of all newborns over a period of 8.5 years, we assumed that our estimates of the incidence of CT in Denmark have low uncertainty, which derives mostly from the sensitivity of the diagnostic approach (in particular detection of IgM antibodies on Guthrie cards by EIA). In the DNNSP, the diagnosis of CT relied on a two-step detection algorithm involving detection of *T*. *gondii*-specific IgM and IgA antibodies on Guthrie cards and confirmatory serology on mother and child. The use of more diagnostic tests and confirmational testing may increase the sensitivity. These estimates rely on the assumption that the incidence of CT was relatively stable after the termination of the survey period and until 2014. Likewise, we assumed that the seroprevalence of *T*. *gondii* was stable in the same period. Data from 1990, when the overall prevalence of *T*. *gondii*-specific IgG antibodies in pregnant women in Denmark was 27% [25] and data from 2014 showing that 23% of 4,571 Danish blood donors (men and women) aged 18–67 years had *T*. *gondii*-specific IgG antibodies (unpublished data) support this assumption. Even though the latter data are not directly comparable because they include a wider age range (and men), they suggest a stability of the seroprevalence in the population.

The major limitations affecting our estimates were the data gaps faced when estimating foetal loss, neonatal death, and seroconversions, and not including all possible health outcomes. For example, we used French data to estimate foetal loss. In France, an active prenatal screening program for CT for pregnant women has been established, which might result in a higher number of voluntary or medical terminations of pregnancy due to CT compared with other countries, including Denmark. In Denmark, voluntary and medical termination of pregnancy due to CT is less likely as most CT cases are not detected during pregnancy. In addition, we were not able to account for seroconversion before weeks 8 to 12 of gestation or seroconversions of the pregnant women where no transplacental transmission of *T*. *gondii* and/or IgG to the child happened. Moreover, because children from the DNFS were only followed until being 12 years of age, we were not able to account for possible sequelae later in life; thus, our estimates might under-estimate the true impact of the disease burden of CT. Additionally, we estimated that some children had more than one sequela, which could potentially lead to a higher disease burden in these cases. We were at this stage unable to account for co-morbidity, which potentially means we under-estimated the overall burden of disease.

The estimated disease burden of CT in Denmark in 2014 amounted to 123 DALYs. This shows that the annual disease burden of CT was substantially lower than the annual disease burden caused by salmonellosis (379 DALYs) and campylobacteriosis (1.586 DALYs) in Denmark [29]. It is however important to highlight that our estimates reflect the burden of CT only, and that the health impact of acquired toxoplasmosis (i.e., post-natal infection) is still unknown and needs to be assessed. Moreover, while the proportion of the disease burden attributable to foodborne transmission varies (61% for CT in low-child-mortality European countries compared with 76% for the other two diseases [1]), the relative contribution of different foods and the effect of different cooking and eating habits remain unknown. Our results highlight the need for further studies to identify the most important sources of *T*. *gondii* infection in order to identify effective pathogen-specific interventions for reducing the burden of disease at a national level.

The estimated disease burden of CT in Denmark is lower than that estimated in The Netherlands. The most recent Dutch study estimated that CT caused 2,251 DALYs annually between 2007 and 2011 [7], resulting in 13.4 DALYs per 100,000 inhabitants, whereas our results show a disease burden of 2.2 DALYs per 100,000 inhabitants. This difference can mostly be explained by the lower incidence of CT in Denmark. In Denmark, the apparent CT incidence of 1.9 per 10,000 live-born children was captured through DNNSP [13], with high coverage over 8.5 years. When comparing the incidence estimates obtained using comparable methods, the incidence of CT in Denmark was almost 11 times lower than in The Netherlands [30], two times lower than in Poland [31], and slightly higher than in Ireland [30]. Likewise, the incidence of CT in countries with low child mortality status, including EU, was estimated by WHO—FERG to be five cases per 10,000 live births, which is three times higher than the incidence observed in Denmark [32].

Differences in the DALY estimates may also reflect differences in input parameters. In particular, we used the recently updated DW for “moderate impairment” (0.031) for chorioretinitis [15], whereas a higher DW (0.08) was used by Havelaar et al. (2007) [18] and Kortbeek et al. (2009) [30]. Applying a DW of 0.08 for chorioretinitis in our model, the estimate increased to 140 (95% UI, 117–167) DALYs per year. Other differences in input parameters were related to life expectancy and incidence of sequelae assumed in the two models.

Since the termination of DNNSP in 2007, zero to five children have been diagnosed with CT annually. Our model estimated that 14 children were born with CT in 2014, and that three children had clinical sequelae of CT in the first year of life; however, no CT cases were registered in 2014; hence, even symptomatic CT cases are missed. These data suggest that CT is currently under-diagnosed or under-reported and that public health surveillance data provide an incomplete picture of the impact of the disease in the country.

## Conclusions

We show that the estimated disease burden and public health impact of CT in Denmark are lower than what has been estimated in other European countries, highlighting the need for country-specific studies. Nevertheless, our study indicates that CT is under-diagnosed or under-reported in Denmark. CT may be a serious and life-long disease, and knowledge on its public health impact is critical to guiding public health policy at the national level.

## Supporting information

S1 Table Scenario Analysis 1–3. The results of Scenario Analysis (SA) 1–3, with inclusion or exclusion of foetal loss (SA1), with varying disability weight (DW) for chorioretinitis (SA2), and with the present or absence of age weighting and/or time discounting at 3% level (SA3), are presented as median years lived with disability (YLDs), years of life lost (YLLs) and Disability Adjusted Life Years (DALYs) per case of congenital toxoplasmosis. The results of the baseline model for estimating the disease burden of congenital toxoplasmosis per case in Denmark in 2014 are highlighted in grey.
(DOCX)
