# Supplementary material for: The disease burden of congenital toxoplasmosis in Denmark, 2014
Source: PLoS One. 2017 May 30;12(5):e0178282. doi: 10.1371/journal.pone.0178282 (PMC5448755; doi:10.1371/journal.pone.0178282)
Supplement: S1 Table — The results of Scenario Analysis (SA) 1–3, with inclusion or exclusion of foetal loss (SA1), with varying disability weight (DW) for chorioretinitis (SA2), and with the present or absence of age weighting and/or time discounting at 3% level (SA3), are presented as median years lived with disability (YLDs), years of life lost (YLLs) and Disability Adjusted Life Years (DALYs) per case of congenital toxoplasmosis. The results of the baseline model for estimating the disease burden of congenital toxoplasmosis per case in Denmark in 2014 are highlighted in grey. (DOCX) [file pone.0178282.s001.docx]

|  | | | | | | | | | | | | | |  |
| --- | --- | --- | --- | --- | --- | --- | --- | --- | --- | --- | --- | --- | --- | --- |
|  | **DW for chorioretinitis**  **(0-0**.**2)** | **No age weighting,**  **No time discounting** | | | **Age weighting,**  **No time discounting** | | | **No age weighting,**  **Time discounting 3%** | | | **Age weighting,**  **Time discounting 3%** | | | |
| **‘Foetal loss’ included** | **Per case** | **YLD** | **YLL** | **DALYs** | **YLD** | **YLL** | **DALYs** | **YLD** | **YLL** | **DALYs** | **YLD** | **YLL** | **DALYs** | |
|  | **0** | 3.33 | 8.89 | 13.88 | 3.56 | 8.89 | 12.44 | 1.22 | 3.00 | 4.33 | 1.33 | 3.22 | 4.67 | |
|  | **0**.**031** | 4.67 | 8.89 | 15.38 | 5.11 | 8.89 | 14.00 | 1.78 | 3.00 | 4.78 | 2.25 | 3.22 | 5.22 | |
|  | **0**.**05** | 5.44 | 8.89 | 16.13 | 5.89 | 8.89 | 14.78 | 2.11 | 3.00 | 5.11 | 2.63 | 3.22 | 5.67 | |
|  | **0**.**1** | 7.44 | 8.89 | 18.50 | 8.22 | 8.89 | 17.11 | 2.89 | 3.00 | 6.00 | 3.75 | 3.22 | 6.56 | |
|  | **0**.**15** | 9.56 | 8.89 | 20.75 | 10.56 | 8.89 | 19.44 | 3.78 | 3.00 | 6.78 | 4.88 | 3.22 | 7.56 | |
|  | **0**.**2** | 11.56 | 8.89 | 23.13 | 12.89 | 8.89 | 21.78 | 4.56 | 3.00 | 7.67 | 6.00 | 3.22 | 8.56 | |
| **‘Foetal loss’ excluded** | **0** | 3.33 | 0.22 | 3.56 | 3.56 | 0.22 | 3.78 | 1.22 | 0.11 | 1.33 | 1.33 | 0.11 | 1.44 | |
|  | **0**.**031** | 4.67 | 0.22 | 4.89 | 5.00 | 0.22 | 5.22 | 1.78 | 0.11 | 1.89 | 2.00 | 0.11 | 2.11 | |
|  | **0**.**05** | 5.33 | 0.22 | 5.67 | 5.89 | 0.22 | 6.11 | 2.11 | 0.11 | 2.11 | 2.33 | 0.11 | 2.44 | |
|  | **0**.**1** | 7.44 | 0.22 | 7.67 | 8.22 | 0.22 | 8.44 | 2.89 | 0.11 | 3.00 | 3.33 | 0.11 | 3.44 | |
|  | **0**.**15** | 9.56 | 0.22 | 9.78 | 10.56 | 0.22 | 10.78 | 3.78 | 0.11 | 3.78 | 4.33 | 0.11 | 4.44 | |
|  | **0**.**2** | 11.56 | 0.22 | 11.78 | 12.89 | 0.22 | 13.11 | 4.56 | 0.11 | 4.67 | 5.33 | 0.11 | 5.44 | |
